# Crosstalk between the Type VI Secretion System and the Expression of Class IV Flagellar Genes in the *Pseudomonas fluorescens* MFE01 Strain

**DOI:** 10.3390/microorganisms8050622

**Published:** 2020-04-25

**Authors:** Mathilde Bouteiller, Mathias Gallique, Yvann Bourigault, Artemis Kosta, Julie Hardouin, Sebastien Massier, Yoan Konto-Ghiorghi, Corinne Barbey, Xavier Latour, Andréa Chane, Marc Feuilloley, Annabelle Merieau

**Affiliations:** 1 LMSM, Laboratoire de Microbiologie Signaux et Microenvironnement, EA 4312, Normandy University, Université de Rouen, 27000 Evreux, France; mathilde.bouteiller7@univ-rouen.fr (M.B.); mathias.gallique@mail.mcgill.ca (M.G.); yvann.bourigault@univ-rouen.fr (Y.B.); yoan.konto-ghiorghi@univ-rouen.fr (Y.K.-G.); corinne.barbey@univ-rouen.fr (C.B.); xavier.latour@univ-rouen.fr (X.L.); andrea.chane1@univ-rouen.fr (A.C.); marc.feuilloley@univ-rouen.fr (M.F.); 2 SFR NORVEGE, Structure Fédérative de Recherche Normandie Végétale, FED 4277, F-76821 Mont-Saint-Aignan, France; 3 Meakins-Christie laboratories, Research Institute of the McGill University Health Centre, Montreal, QC H4A 3J1, Canada; 4 Department of Chemical Engineering, McGill University, Montreal, QC H3A 0C5, Canada; 5 Plateforme de Microscopie de l’Institut de Microbiologie de la Méditerranée, IMM, Institut de Microbiologie, FR3479, Campus CNRS, 13402 Marseille cedex 20, France; akosta@imm.cnrs.fr; 6 Polymers, Biopolymers, Surface Laboratory, UMR 6270 CNRS, University of Rouen, F-76821 Mont-Saint-Aignan cedex, France; julie.hardouin@univ-rouen.fr (J.H.); sebastien.massier@univ-rouen.fr (S.M.); 7 PISSARO Proteomics Facility, Université de Rouen, F-76821 Mont-Saint-Aignan, France

**Keywords:** type VI secretion system, flagella, FliA, FlgM, Hcp1, motility inhibition, *Pseudomonas fluorescens* MFE01

## Abstract

Type VI secretion systems (T6SSs) are contractile bacterial multiprotein nanomachines that enable the injection of toxic effectors into prey cells. The *Pseudomonas fluorescens* MFE01 strain has T6SS antibacterial activity and can immobilise competitive bacteria through the T6SS. Hcp1 (hemolysin co-regulated protein 1), a constituent of the T6SS inner tube, is involved in such prey cell inhibition of motility. Paradoxically, disruption of the *hcp1* or T6SS contractile tail *tssC* genes results in the loss of the mucoid and motile phenotypes in MFE01. Here, we focused on the relationship between T6SS and flagella-associated motility. Electron microscopy revealed the absence of flagellar filaments for MFE01Δ*hcp1* and MFE01Δ*tssC* mutants. Transcriptomic analysis showed a reduction in the transcription of class IV flagellar genes in these T6SS mutants. However, transcription of *fliA*, the gene encoding the class IV flagellar sigma factor, was unaffected. Over-expression of *fliA* restored the motile and mucoid phenotypes in both MFE01Δ*hcp1*+*fliA*, and MFE01Δ*tssC*+*fliA* and a *fliA* mutant displayed the same phenotypes as MFE01Δ*hcp1* and MFE01Δ*tssC*. Moreover, the FliA anti-sigma factor FlgM was not secreted in the T6SS mutants, and *flgM* over-expression reduced both motility and mucoidy. This study provides arguments to unravel the crosstalk between T6SS and motility.

## 1. Introduction

Environmental strains must adapt to and conquer ecological niches to survive. During this territorial conflict, secretion apparatuses are essential for such competition and adaptation. Among these mechanisms, the Type VI Secretion System (T6SS), an inverted bacteriophage-like structure, allows bacteria to defend against or attack target cells using various secreted effectors [1,2]. T6SS confers a competitive advantage to the bacteria producing them by killing target bacteria [3,4,5], recognising isogenic cells [6,7,8], or immobilising adjacent antagonistic cells [9]. T6SS is also involved in the uptake of metal ions, such as iron, zinc, and manganese [10,11,12,13,14], and likely bacterial communication [15]. The T6SS apparatus is composed of at least 13 essential conserved proteins (named TssA to TssM, for type six secretion), which constitute the “core component” [16,17]. The membrane complex TssJLM anchors T6SS to the bacterial envelope and positions the baseplate complex [18]. This structure allows proper contractile tail assembly and may initiate sheath contraction, similarly to bacteriophage. Contraction of the sheath surrounding the inner-Hcp protein tube propels effectors into prey cells or the extracellular medium. A ClpV ATPase then recycles the sheath subunits for new firing, and the membrane complex can be used for new T6SS assembly [18].

Another key factor in bacterial competition is the ability to move and colonise environmental niches. Fluorescent *Pseudomonas* are considered to be excellent colonisers of plant rhizospheres because their densities and activities are higher near the roots than in bulk soil [19,20]. Numerous *Pseudomonas spp*. are considered to be plant growth-promoting rhizobacteria (PGPR), which protect plant roots from phytopathogens and are beneficial for root growth [21]. Their fitness and biocontrol efficiencies are often associated with bacterial motility, chemotaxis, and direct antagonism mediated by the synthesis of toxic agents [22]. These properties are closely associated with the presence of bacterial extracellular appendages, such as flagella and secretion systems [23,24]. As the root system disseminates several exudates that provide a rich source of nutrient, the deployment of flagella is crucial for an earlier colonisation both for PGPR [25,26,27] and phytopathogenic *Pseudomonas* [28].

Flagella assembly requires the ordered export of thousands of structural subunits across the cell membrane and is achieved by a type III export machinery located at the base of the flagellum [29]. The promoters that control expression of flagellar genes have been divided into three classes for enterobacteria [30], whereas in *Pseudomonas*, flagellar gene expression is controlled in a four-tiered hierarchy of transcriptional regulation (called classes I to IV) [31]. Transcriptional regulators, including RpoN, FliA, FleR, and FleQ, finely control expression of the flagellar genes needed for the coordinated assembly of flagella [32].

We previously described the *Pseudomonas fluorescens* MFE01 strain, which secretes large amounts of Hcp proteins, a marker of T6SS functionality [33]. MFE01 has antibacterial activity against a wide range of competitor bacteria associated with the T6SS. Genomic analysis showed the existence of a unique T6SS core component locus and at least three orphan *hcp* genes, named *hcp1*, *hcp2*, and *hcp3* [34]. The Hcp2 and Hcp3 proteins are directly involved in the killing activity of MFE01, whereas Hcp1 proteins of MFE01 are essential for inhibiting the motility of prey cells [9,33,34]. Indeed, MFE01 can sequester prey bacteria under swimming and swarming conditions, whereas the *hcp1* mutant of MFE01, MFE01Δ*hcp1*, cannot. Surprisingly, mutation of *hcp1* has pleiotropic effects on the phenotype of MFE01, affecting its mucoidy and motility [9]. Therefore, this study focuses on the close ties between the T6SS and flagellar filament synthesis in *P. fluorescens* MFE01.

## 2. Materials and Methods

### 2.1. Bacterial Strains, Plasmids, and Culture Conditions

All the strains and plasmids used in this study are listed in Table 1. All strains were grown in LB (Luria Bertani) medium with shaking (180 rpm). *P. fluorescens* strains were grown at 28 °C and *Escherichia. coli* strains at 37 °C. Media were supplemented with antibiotics, as appropriate: 15 µg/mL tetracycline (*P. fluorescens*), 15 µg/mL (*E. coli*) or 50 µg/mL (*P. fluorescens*) gentamycin, 50 µg/mL (*E. coli*) or 100 µg/mL (*P. fluorescens*) kanamycin. The cultures of strains carrying pPSV35 plasmid were supplemented with 100 µg/mL IPTG and strains containing the pJN105 plasmid with 1% arabinose for gene expression. 

### 2.2. Mucoid Phenotype and Swimming Motility

Mucoid and swimming assays were performed as described by Decoin et al. [33]. Briefly, strains were plated on 1.5% LB agar and incubated for 24 h at 28 °C before assessment of the mucoid phenotype. To observe swimming motility, strains were grown overnight in LB media and centrifuged at 7500· *g* for 5 min at room temperature. Biomass was spotted onto 0.3% LB-agar plates with a sterile toothpick and incubated at 28 °C overnight before measuring the “swimming” diameters.

### 2.3. Disruption of the fliA Gene in P. Fluorescens MFE01 Strain

PCR was performed under standard conditions using Phusion® High-Fidelity DNA polymerase (NEB). The temperature of primer hybridisation was calculated using NEB Tm calculator (https://tmcalculator.neb.com/#!/main). The *fliA* in-frame disruption, obtained by the central deletion of 411 bp, was achieved by PCR using muta1-fliA-F/muta2-fliA-EcoRI-R primers (for amplicon A: 671 bp) and muta3-fliA-EcoRI-F/muta4-fliA-R primers (for amplicon B, 723 bp product) (Table 2). The PCR products corresponded to the upstream and downstream parts of the MFE01 *fliA* gene, each carrying an *EcoRI* restriction site. Both A and B amplicons were digested by *EcoRI* (NEB) and ligated with T4 DNA ligase (NEB). A third PCR was then carried out with muta1-fliA-F/muta4-fliA-R primers. The resulting disrupted *fliA* construct was introduced into the pAKE604 suicide vector, previously digested by *SmaI* (blunt-ended) (NEB), and ligated with T4 DNA ligase (NEB) [38]. This construction was verified by sequencing and introduced into the *E. coli* S17.1 strain [36]. The recombinant plasmid was transferred by biparental mating: recipient MFE01 and the S17.1 strain containing pAKE604Δ*fliA* were mixed at a 1:1 ratio and spotted onto LB-agar medium and incubated at 37 °C overnight. The biomass mixture was resuspended in 1 mL sterile saline solution and 0.1 mL of the cell suspension spread on LB-agar plates supplemented with rifampicin 50 µg/mL (for MFE01 selection and *E. coli* S17.1 killing) and kanamycin 100 µg/mL (to select cells containing recombinant plasmid) and incubated at 28 °C for 48 h. Colonies were isolated on LB-agar plates supplemented with 10% sucrose to select the second homologous recombinants. The resulting *fliA* mutant was verified by DNA sequencing and named MFE01Δ*fliA*.

### 2.4. Construction of the Revertant Strain MFE01ΔtssC-R

PCR was performed under standard conditions using Phusion® High-Fidelity DNA polymerase (NEB). The *tssC* amplicon (wild-type gene and environment) was amplified from *P*. *fluorescens* strain MFE01 with muta1-tssC-F/muta4-tssC-R primers (Table 2). The primer hybridisation temperature was calculated with NEB Tm calculator. The resulting *tssC* amplicon was introduced into the pAKE604 suicide vector, previously digested by *SmaI* (blunt-ended) (NEB), and ligated with T4 DNA ligase. The construct was verified by DNA sequencing and then introduced into *E. coli* S17.1. The recombinant plasmid was transferred by biparental mating: the recipient MFE01Δ*tssC* and the S17.1 strain containing pAKE604-*tssC* were mixed at the same ratio and spotted onto LB agar medium and incubated at 37 °C overnight. The biomass mixture was resuspended in 1 mL sterile saline solution and 0.1 mL of the cell suspension spread on LB-agar plates supplemented with rifampicin 50 µg/mL (for MFE01Δ*tssC* selection and *E. coli* S17.1 killing) and kanamycin 100 µg/mL (to select cells containing the recombinant plasmid) and incubated at 28 °C for 48 h. Colonies were isolated on LB-agar plates supplemented with 10% sucrose to select the second homologous recombinants. The resulting revertant strain was verified by DNA sequencing and named MFE01Δ*tssC*-R.

### 2.5. Translational Fusion of Flag Sequence into the MFE01 flgM Gene

A *flag* sequence was introduced in the 3′ region of the *flgM* gene. PCR was performed using Phusion® High-Fidelity DNA polymerase (NEB) under standard conditions. The temperature of primers hybridisation was calculated with the NEB Tm calculator. The amplicon A corresponded to the sequence upstream of the stop codon of the *flgM* gene and amplicon B to the downstream region. Muta1-3′flag-flgM/Muta2-3′flag-flgM primers were used to obtain amplicon A (709 pb) and Muta3-3′flag-flgM/Muta4-3′flag-flgM to obtain amplicon B (740 pb). An overlapping-PCR using the 3′flag sequence of amplicon A and 5′flag sequence of amplicon B was performed with the M1-3′flag-flgM and M4-3′flag-flgM primers. The resulting amplicon, containing the *flgM::flag* construct, was then inserted into the pAKE604 suicide vector, previously digested by *SmaI* (NEB), and ligated with T4-DNA-ligase (NEB). This construct was verified by sequencing and introduced into the *E. coli* S17.1 strain. The recombinant plasmid was transferred by biparental mating: recipient MFE01 or MFE01Δ*hcp1* and the S17.1 strain containing pAKE604-*flgM::flag* were mixed at a 1:1 ratio and spotted onto LB-agar medium and incubated at 37 °C overnight. The biomass mixture was resuspended in 1 mL sterile saline solution and 0.1 mL of the cell suspension spread on LB-agar plates supplemented with 50 µg/mL rifampicin (for MFE01 selection and *E. coli* S17.1 killing), 100 µg/mL kanamycin (to select cells containing recombinant plasmid) and 15 µg/mL tetracycline (to select the *hcp1* deletion mutant) and incubated at 28 °C for 48 h. Colonies were isolated on LB-agar plates supplemented with 10% sucrose and 15µg/mL tetracycline for MFE01Δ*hcp1* to select the second homologous recombinants. The resulting Flag insertion was verified by a western-blot experiment, using anti-Flag antibody coupled to alkaline phosphatase (Sigma-Aldrich, St. Louis, MO, USA).

### 2.6. Insertion of fliA and flgM into Expression Vectors

The fliA-EcoRI-F/fliA-XbaI-R primers and flgM-EcoRI-F/flgM-XbaI-R primers (Table 2) were used to amplify the *fliA* and *flgM* genes, respectively, using Phusion® High-Fidelity DNA Polymerase (NEB). PCR was performed under standard conditions. The primer hybridisation temperatures were calculated with NEB Tm calculator. The amplified fragment and the pPSV35 shuttle vector [37] or pJN105 shuttle vector [39] were digested with *Eco*RI and *Xba*I (NEB) to generate cohesive ends. The coding region of the *fliA* gene was inserted into pPSV35 downstream of the PlacUV5 promoter and the coding region of the *flgM* gene into pJN105 downstream of the arabinose inducible promoter using T4-DNA-ligase (NEB). The resulting plasmids, pPSV35-*fliA* and pJN105-*flgM*, were used to transform *E. coli* Top10® cells by thermal shock. Plasmid DNA was isolated using the GeneJET Plasmid Miniprep Kit (ThermoFisher Scientific, Waltham, MA, USA) and verified by PCR with plasmid-specific primers and DNA sequencing.

### 2.7. Introduction of Plasmid into MFE01, MFE01 Mutants or MFN1032

Fresh colonies of MFE01, MFE01 mutants or MFN1032 [35] were washed twice with 1 mL of cold sterile water and resuspended in 100 μL of cold sterile water. One hundred nanograms of plasmid (pPSV35, pJN105 or derivatives) were added and electroporation performed in 1-mm electroporation cells at 1.8 kV for 5 ms (GTF100 Gene Transformer, Savant Inc., New York, NY, USA). LB was added and the mixture incubated at 28 °C for 1 h with shaking (180 rpm). Transformed bacteria were then selected by plating on LB-agar supplemented with gentamycin. 

### 2.8. Putative FliA Promoters and Consensus Motif

The consensus motif (TAAAGTTT-N11-GCCGATAA), corresponding to promoter sequences recognised by FliA [40], was used to search for promoter sequences recognised by FliA upstream of the flagellar genes in MFE01. The sequence logo, corresponding to putative FliA-dependant promoters in the MFE01 strain, was generated using Multiple Em for Motif Elicitation (MEME: http://meme-suite.org/tools/meme). GenBank accession numbers: *flaA*-to-*fliT* region, MT018347; *flgM*-region, MT018348; *motA*-*motB*-region, MT018349

### 2.9. Extraction of Total RNA from P. Fluorescens MFE01

Total RNA was extracted using the hot acid-phenol protocol described by Bouffartigues et al. [41], with modifications. Bacteria were lysed in lysis buffer (0.02 M sodium acetate, pH 5.5, 0.5% (*w*/*v*) SDS, 1 mM EDTA) in early exponential growth (OD_580nm_ = 1). An acid-phenol solution (Sigma-Aldrich) was heated to 60 °C. The lysate was mixed with an acid-phenol/water solution (5:1, *v*/*v*) and incubated at 60 °C for 3 min. The aqueous phase was then removed after centrifugation at 13,000· *g* for 5 min and mixed two times with the acid-phenol/water solution (5:1, *v*/*v*) and once with chloroform/isoamyl alcohol 24:1 (Sigma-Aldrich, St. Louis, USA). Total RNA from the aqueous phase was precipitated overnight at -20 °C with 100% ethanol (2∶1, *v*/*v*) containing 1 M sodium acetate (1∶10, *v*/*v*). A centrifugation at 13,000· *g* for 30 min at 4 °C was performed to remove the supernatant. The RNA pellet was then washed twice with 70% ethanol, dried at room temperature for 45 min, and dissolved in RNase-free water. DNA was digested with Turbo^TM^ DNase (ThermoFisher Scientific, Waltham, Massachusetts, USA) and the DNase was inactivated by incubating at 75 °C for 10 min after adding 0.01 M EDTA. The absence of DNA was verified by PCR using qRT-PCR-RecA-F/qRT-PCR-RecA-R primers. RNA extraction was verified on a 2% agarose gel and the concentration determined by measuring the OD ratios A_260_/A_280_ and A_260_/A_230_ by Nanodrop.

### 2.10. cDNA Amplification

Total cDNA was amplified from total RNA extracts with the High-Capacity cDNA Reverse Transcription Kit (Applied Biosystems, Waltham, MA, USA) according to the manufacturer’s recommendations. The 50-µL reaction, containing 25 ng of RNA, was incubated at 25 °C for 10 min, 37 °C for 2 h, and inactivated for 5 min at 85 °C.

### 2.11. Quantitative Reverse Transcription-PCR

qRT-PCR was performed using the protocol described by Guyard-Nicodème et al. [42]. All samples were analysed independently at least six times. The primers (Table 2) were designed using Primer Express 3 software. A single PCR product for each primer pair was verified prior to use. The 13-µL reactions were performed using the following conditions: 6.5 µL SYBR Green PCR Master Mix (Applied Biosystems), 0.3 µM primers, and 3 µL cDNA. PCR reactions were performed with the 7500 Real Time PCR System apparatus (Applied Biosystems, Waltham, MA, USA). Relative quantification of the mRNAs of interest was obtained by the comparative CT (2^−ΔΔCT^) method, using MFE01 *recA* mRNA as an endogenous control. The relative quantification (RQ) of mRNA was calculated using the 2^−ΔΔCT^ method between the wild-type strain containing the empty vector (+EV) and the mutant strains containing the empty vector (+EV). Non-parametric Mann–Whitney Tests (two tailed) were used for statistical analyses. A *p*-value < 0.05 was considered to be statistically significant.

### 2.12. Supernatant Protein Extraction

Overnight cultures (25 mL) were centrifuged at 7500× *g* for 5 min at room temperature. The supernatants were filtered through a Millipore membrane with 0.22-µm pores (Merck). Trichloroacetic acid (TCA) (Sigma-Aldrich, St. Louis, USA) was added to a final concentration of 10% and the mix incubated overnight at 4 °C. The supernatant was removed by centrifugation at 13,000× *g* for 30 min at 4 °C. The pellet was washed twice with 5 mL cold 100% acetone (Merck) (without resuspending the pellet) and centrifuged at 13,000× *g*, for 30 min at 4 °C. The protein pellet was air dried for 30 min at 4 °C.

### 2.13. Intracellular Protein Extraction

Overnight cultures (25 mL) were centrifuged at 7500× *g* for 5 min at room temperature. The pellet was washed three times with 5 mL saline solution before resuspending in 20 mM Tris-HCl buffer (pH 7.4). Protease inhibitor (cOmplete™, EDTA-free Protease Inhibitor Cocktail, Krackeler Scientific, Albany, NY, USA) was added, according to the manufacturer’s recommendations and the bacteria were lysed by sonication using a Branson Digital Sonifier®, 50% amplitude, three cycles of 1 min, alternation of 1 s sonication, and 1 s break, on ice. The intracellular content was recovered after centrifugation at 8000× *g* for 10 min at 4 °C and treatment with Benzonase® nuclease (Sigma-Aldrich, St. Louis, MO, USA), according to the manufacturer’s recommendations. For some western-blot experiments, the proteins were concentrated using centrifugal filter units (Merck, Darmstadt, Germany) at 5000× *g* for 45 min with a 10, 30, or 100 kDa cutoff.

### 2.14. SDS-PAGE Analysis

Proteins were resuspended (supernatant proteins) or mixed (intracellular proteins) with 2X Laemmli sample buffer containing 5% β-mercapto-ethanol before incubation for 5 min at 100 °C. The proteins corresponding to 2.5 mL of culture (supernatant proteins), 0.25 mL of culture (intracellular proteins) or concentrated fractions (intracellular proteins), were separated on a 12% or 15% SDS-PAGE gel and Coomassie Blue used to visualise the proteins. Images were captured using a GS-800 densitometer (Bio-Rad, Hercules, CA, USA).

### 2.15. Western-Blot Analysis

The proteins separated on 15% SDS-PAGE gel were transferred to a nitrocellulose membrane using the Invitrogen™ iBlot™ 2 system (P0 program). Nitrocellulose membranes were then incubated 1 h in blocking buffer (1X TBS with 5% skim milk). Membranes were washed three times with 1X TBS containing 0.1% Tween®20 Sigma-Aldrich, St. Louis, MO, USA) before incubation with anti-Flag antibodies coupled to alkaline phosphatase (Monoclonal ANTI-FLAG® M2-Alkaline Phosphatase antibody produced in mouse, Sigma-Aldrich, St. Louis, MO, USA). Membranes were washed three times in 1X TBS containing 0.1% Tween®20, and the alkaline phosphatase conjugate substrate kit (Bio-Rad, Hercules, CA, USA) was used to visualise the presence of anti-Flag antibodies.

### 2.16. Protein Identification by nanoLC-MS/MS

The analysis was performed on five biological replicates of MFE01 and MFE01*Δhcp1* supernatants, as previously described [43]. Briefly, the supernatant protein pellet was resuspended in R2D2 buffer (7M Urea, 2M Thiourea, 5mM TBP (tri-n-butylphosphine), 20mM DTT, 0,5% C7BzO and 2% CHAPS). The sample was mixed with SDS-loading buffer 2X (63 mM Tris-HCl, pH 6.8, 10 mM DTT, 2% SDS, 0.02% Bromophenol Blue, and 10% glycerol) and loaded onto a SDS-PAGE stacking gel (7%). After a short electrophoresis (10mA, 15 min), the gel was stained with Coomassie blue and destained with a solution containing 50% ethanol, 10% acetic acid, and 40% deionised water. The band containing the proteins was excised, washed with water. Proteins were alkylated with 15 mM iodoacetamide for 45 min in the dark before submitted to trypsin digestion (1 µg per band), overnight at 37 °C with shaking. Peptide extraction was carried out 3 times with 100% ACN. Peptides were then dried completely using a Speedvac concentrator (SPD111V, Thermo Fisher Scientific) and stored at −20 °C. Peptides were then analysed by tandem mass spectrometry using an LTQ-Orbitrap Elite mass spectrometer coupled to an Easy nLC II system (both Thermo Scientific). Raw data files were first processed using Proteome Discoverer 1.4 software (Thermo Scientific). Peak lists were searched using the MASCOT search software (Matrix Science) against the database Pseudomonas sp. B10 (www.pseudomonas.com). Database searches were performed with the following parameters: 2 missed trypsin cleavage sites allowed; variable modifications: carbamidomethylation on cystein, and oxidation on methionine. The parent-ion and daughter-ion tolerances were 5 ppm and 0.35 Da, respectively.

### 2.17. Flagellin and Hcp Proteins Mass Spectrometry Identification from SDS-PAGE

Mass spectroscopy (MS) analyses were performed with a MALDI-TOF AutoflexIII (Brucker) in positive ion mode, as previously described [9]. Statistical analyses of the sequences involved determining the probability based on the Mowse score with MASCOT software (peptide tolerance = 100 ppm and mass values = MH^+^). A *p*-value of < 0.05 was considered significant. The criteria used to accept a protein identification based on peptide mass fingerprinting (PMF) data included a probability score greater than a threshold score defined by the MASCOT software (www.matrixscience.com/search_form_select.html).

### 2.18. Transmission Electron Microscopy

Bacteria were grown on LB medium at 28 °C for 48 h. Microscopy negative staining was performed as follows: 5-μL drops of the bacterial suspension were placed directly on glow-discharged carbon-coated grids (EMS) for 3 min. The grids were then washed with two drops of 2% aqueous uranyl acetate and stained with a third drop for 1 min. Grids were dried on filter paper, and the samples were analysed using a Tecnai 200 KV electron microscope (FEI) and digital acquisition was performed with a numeric camera (Eagle, FEI). The average number of flagella per bacterium was determined after observation of at least 35 bacteria for each strain.

## 3. Results and Discussion

### 3.1. MFE01 Motility and Mucoidy are Specifically Dependent on Hcp1

**Figure 1:**
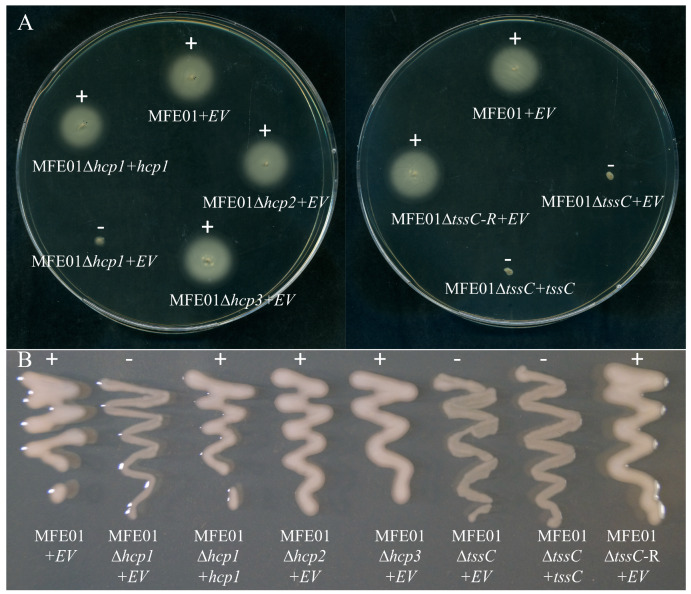
Swimming motility and mucoidy of *P. fluorescens* MFE01 and mutants. (**A**) “Swimming motility”. Swimming assays were performed on 0.3% LB-agar, supplemented with 50 µg/mL gentamycin, overnight at 28 °C. EV: empty pPSV35 vector, +: motile, −: non-motile. The images shown are representative of three assays (*n* = 3). (**B**) Mucoid phenotype. Mucoidy was assessed on 1.5% LB agar, supplemented with 50 µg/mL gentamycin, after incubation for 24 h at 28 °C. EV: empty pPSV35 vector, +: mucoid, −: non-mucoid. The images shown are representative of three assays (*n* = 3).

We studied motility on 0.3% LB-agar medium and mucoidy of various MFE01 mutants. Disruption of the *hcp1* gene resulted in the loss of both mucoid and motile phenotypes, as previously described, which were restored by the in *trans* introduction of *hcp1* into MFE01Δ*hcp1* [9]. Similarly, the MFE01Δ*tssC* strain, in which T6SS is non-functional by contractile tail inactivation, was non-motile under swimming conditions and non-mucoid, whereas MFE01Δ*hcp2* and MFE01Δ*hcp3* exhibited wild-type phenotypes (motile and mucoid) (Figure 1).

However, insertion of the *tssC* gene in *trans* in MFE01Δ*tssC*, MFE01Δ*tssC*+*tssC*, restored neither the motile nor mucoid phenotypes (Figure 1). Thus, the expression of *tss*C in *trans* in MFE01Δ*tssC* appears to be insufficient to properly restore T6SS functionality. We verified the absence of another mutation outside the *tssC* gene in MFE01Δ*tssC* by reintroducing the native *tssC* gene at its usual chromosomal location to obtain the revertant strain MFE01Δ*tssC*-R. MFE01Δ*tssC*-R was motile and mucoid, demonstrating that an unidentified mutation was not responsible for the complementation failure and that introduction of the native *tssC* gene restores the motile and mucoid phenotypes (Figure 1). Overall, these results highlight a putative specific link between Hcp1-related T6SS (Hcp1-T6SS), motility, and mucoidy.

Inactivation of the T6SS apparatus has already been shown to correlate with the loss of motility of enterobacteria, such as the *Escherichia coli* APEC SEPT362 and *Citrobacter freundii* CF74 strains [44,45]. The authors of the *E. coli* APEC study concluded that TssM (a T6SS membrane-associated protein with ATPase activity, named in this study as IcmF) is essential for bacterial motility and affects expression of the flagella regulon, but did not provide an explanation for this mechanism [45]. Liu et al. observed that T6SS mutations, particularly a mutation in the *hcp2* gene, disturbs the flagellar system at the transcriptional level [44]. The mechanism responsible for the loss of motility of these two strains was not determined. In the *P. aeruginosa* PAO1 strain, mutation of the *icmF3* gene, corresponding to the *tssM* gene related to H3-T6SS, provoked defects in swimming motility without decreased expression of the flagella regulon [45]. In *Ralstonia solanacearum*, a species related to the *Pseudomonas* genus, it was demonstrated that a *tssB* gene mutation is detrimental for the expression of the flagella regulon, leading to the loss of motility [46].

### 3.2. Disruption of the hcp1 Gene Results in the Lack of Flagella

**Figure 2:**
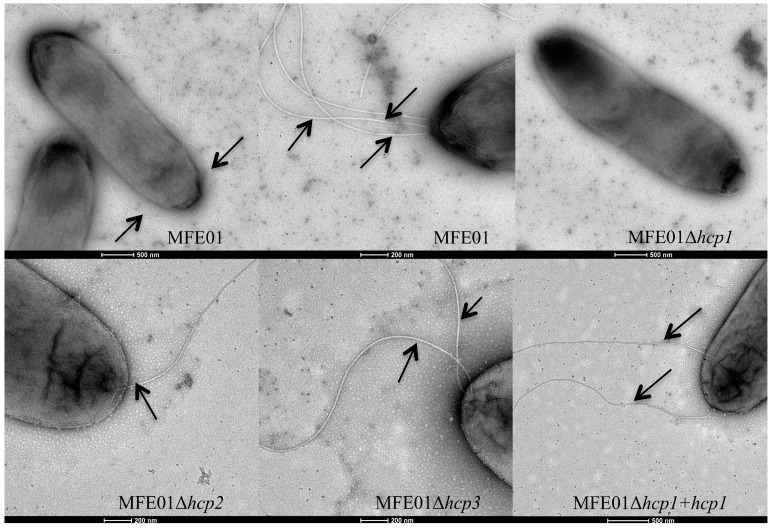
Flagella of *P. fluorescens* MFE01 and derivatives. Transmission electron microscopy images of bacteria grown at 28 °C (negative stain). All tested strains (MFE01, MFE01Δ*hcp1*, MFE01Δ*hcp2*, and MFE01Δ*hcp3*) contained the empty pPSV35 vector. Arrows indicate a flagellum. These images are representative of at least 35 bacteria for each strain.

The motility of *Pseudomonas* is dependent on the synthesis of flagella and is controlled by chemotaxis and flagellar proton-channel proteins, which control flagellar activity and rotation [47,48]. We examined various mutants by transmission electron microscopy to understand the loss of motility of MFE01Δ*hcp1* (Figure 2). The MFE01 wild-type strain had a mean of two polar flagella, with up to three polar flagella. Disruption of *hcp2* or *hcp3*, which resulted in motile strains*,* did not affect the mean number of flagella, whereas MFE01Δ*hcp1*, which was non-motile, had no flagella. By contrast, the motile MFE01Δ*hcp1+hcp1* strain had the same mean number of flagella as MFE01. Thus, the lack of motility of MFE01Δ*hcp1* is due to a defect in flagellar filament synthesis.

### 3.3. FliA Controls Motility and the Mucoid Phenotype

Flagellar biosynthesis is regulated differently in various *Pseudomonas* species [49]. In *P. aeruginosa*, it has already been demonstrated that the sigma factor FliA, also called sigma factor σ28, controls the expression of genes in several functional categories, including chemotaxis, motility, and attachment, as well as secreted factors that are alginate responsive for mucoidy [50]. 

**Figure 3:**
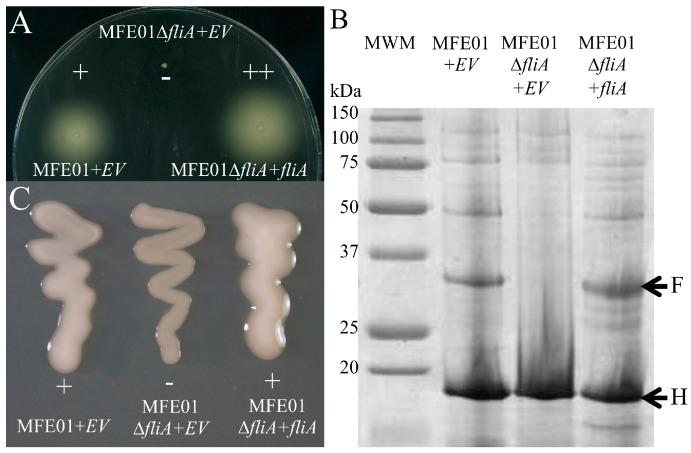
Effect of *fliA* mutation on phenotypes of MFE01. (**A**) Swimming motility. Swimming assays were performed on 0.3% LB-agar for 24 h at 28 °C. +: motile, −: non-motile, ++: hyper-motile. EV: empty pPSV35. The images shown are representative of three assays (*n* = 3). (**B**) Hcp and flagellin secretion. Concentrated supernatants of cultures in the late exponential growth phase, grown at 28 °C, were analysed by SDS-PAGE (12% separation gel) and Coomassie staining. Bands indicated by the arrows and labelled H and F were identified by MALDI/ToF as Hcp and flagellin proteins, respectively. The periplasmic alkaline phosphatase of MFE01, used as control for cell lysis, was not detected in these experiments. MWM: molecular weight marker, EV: empty pPSV35 vector. (**C**) Mucoid phenotypes. Mucoidy was assessed on 1.5% LB-agar, supplemented with 50 µg/mL gentamycin, after incubation for 24 h at 28 °C. EV: empty pPSV35 vector, +: mucoid, −: non-mucoid. The images shown are representative of three assays (*n* = 3).

We next focused on the phenotypic effects of *fliA* gene disruption in the MFE01 strain. As expected, MFE01Δ*fliA* was non-motile and motility was restored after the reintroduction of *fliA* in *trans* in this mutant, giving the MFE01Δ*fliA*+*fliA* strain (Figure 3A). Moreover, supernatants of MFE01Δ*fliA+fliA* showed flagellin secretion, whereas supernatants of MFE01Δ*fliA* did not (Figure 3B), without affecting Hcp protein secretion, the hallmark of T6SS functionality. Deletion of *fliA* also affected the mucoid phenotype (Figure 3C), suggesting that FliA regulates the expression of mucoidy-associated genes in the MFE01 strain.

### 3.4. FliA Activates the Transcription of Flagellar Class IV Genes in the MFE01 Strain

In *Pseudomonas aeruginosa* PAO1, FleQ is a transcriptional activator required for class I and II flagellar genes expression, and RpoN controls the transition from class II to class III flagellar genes expression [31]. In 2018, Blanco-Romero et al. ascertained that the transcriptional regulator FleQ is the master regulator of the flagellar cascade in *Pseudomonas* because *fleQ* mutants of *P. aeruginosa*, *P. putida,* and *P. fluorescens* are non-motile and lack flagella [29,47,51,52]. Furthermore, they demonstrated by ChlP analysis that FleQ is a global regulator of motility-related genes and exopolysaccharides production in *P. fluorescens* F113 and *P. putida* KT2440. Finally, FliA acts as a checkpoint, permitting the transition of flagella from class III (hook-based-body) to class IV (filament) (see Figure S1 for details and Table S1 for genes function and MFE01 genes GenBank accession numbers) [29,47,51,52]. 

The sigma factor FliA binds to RNA polymerase and recognises sites upstream of target genes. Thus, FliA specifically activates the expression of class IV flagellar genes located downstream of specific promoters. FliA allows the control of flagellar filament assembly by regulating, for example, the expression of genes encoding flagellin (*flaA* or *fliC*), the chaperone FlgN, anti-sigma FlgM, and MotA and MotB (proton-driven flagellar motor). In contrast, in *P. aeruginosa*, the transcription of genes encoding the flagellin export chaperone FliS and capping protein FliD, which control flagellin assembly, is not controlled by FliA but by both RpoN and FleQ [53]. In the *P. putida* KT2440 strain, FliS and FliD are produced under the control of FliA [54]. These examples highlight the differences in regulation of FliS, FliD, and FliT production by *P. aeruginosa* and *P. putida*. In the *P. fluorescens* F113 strain, Redondo-Nieto et al. studied the transcriptional organisation of the region involved in the synthesis of the flagellar filament [49]. They concluded that *fliC* (*flaA*) transcription is regulated both by FliA and RpoN /FleQ. It has been demonstrated that FliA controls the expression of *flgZ*, encoding FlgZ, which interacts with the stator protein in the F113 strain [55]. In this study, the authors found no putative *fliA* promoters upstream of the *fliS* or *fliT* gene. 

**Figure 4:**
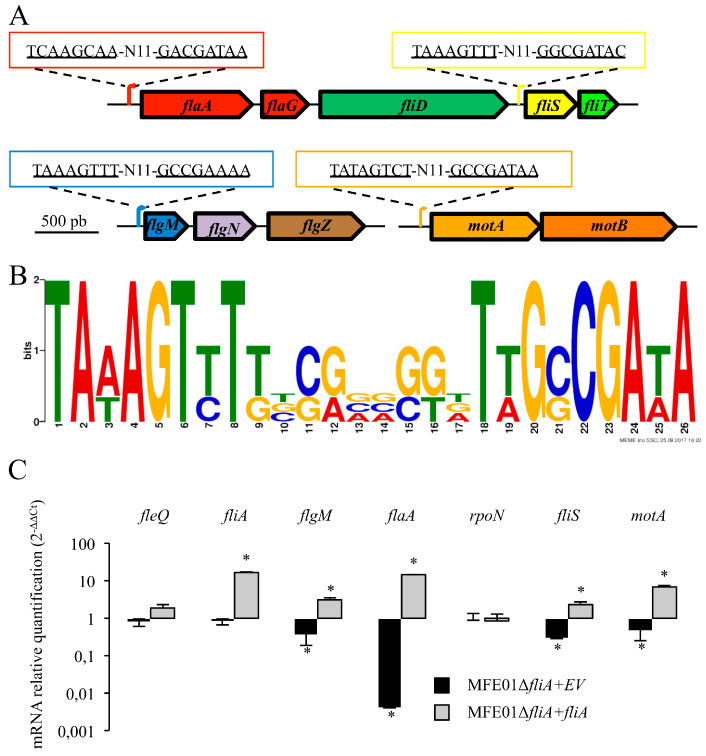
Transcription of flagellar genes in MFE01 and derivatives. (**A**) Putative promoters upstream of flagellar genes. A consensus motif (TAAAGTTT-N11-GCCGATAA), corresponding to promoters recognised by FliA in *Pseudomonas aruginosa* [40], was used to search for putative FliA-dependent promoters upstream of flagellar genes in MFE01. Sequences corresponding to putative promoters are indicated. (**B**) MEME sequence logo of the four putative promoter motifs. The sequence logo, generated by Multiple Em for Motif Elicitation (MEME), corresponding to the putative FliA-dependent promoters, is shown. (**C**) Flagellar gene expression in MFE01Δ*fliA+EV* and MFE01Δ*fliA+fliA*. The reported mRNA levels are relative to those obtained in MFE01 carrying the empty pPSV35 vector. Relative levels of gene expression are based on the comparative CT (2^–ΔΔCT^) method, using MFE01 *recA* mRNA as endogenous control. Statistical analyses were performed using Non-parametric Mann–Whitney Tests (two tailed). A *p*-value < 0.05 was considered to be statistically significant. *P < 0.05, *n* = 6. Data shown represent the mean ± SEM. In this experiment, the primers used for *fliA* were located downstream of the *fliA* central in-frame deletion.

Thus, we used the consensus motif (TAAAGTTT-N11-GCCGATAA), corresponding to promoters recognised by FliA in *P. aeruginosa* [40], to search for putative promoters upstream of the flagellar genes in MFE01. Sequences partially corresponding to putative consensus sequences of FliA promoters were found upstream of the *flaA*, *fliS*, *flgM,* and *motA* genes (Figure 4A). A sequence logo corresponding to the four putative promoters, generated using Multiple Em for Motif Elicitation (MEME), is shown in Figure 4B. We studied the relative expression of several flagellar genes between MFE01 and MFE01Δ*fliA* by qRT-PCR to elucidate FliA-dependant gene transcription in *P. fluorescens* MFE01. We measured the impact of FliA on the transcription of flagellar genes located downstream of the putative FliA-dependent promoters and on *fleQ*, *rpoN,* and *fliA* genes transcription. Disruption of the *fliA* gene significantly decreased the transcription of *flgM*, *flaA*, *fliS*, and *motA* (Figure 4C), without affecting *fleQ* or *rpoN* transcription, confirming the FliA specificity of identified promoters. In this experiment, the primers used for *fliA* were located downstream of the *fliA* central in-frame deletion, explaining the absence of a decrease in *fliA* transcription in MFE01Δ*fliA*. This shows that FliA is not required for *fliA* gene expression, suggesting the absence of a positive feedback regulation. Introduction of *fliA* in MFE01Δ*fliA*, resulting in the MFE01Δ*fliA*+*fliA* strain, except for the *rpoN* and *fleQ* genes, significantly increased the expression of the tested genes relative to the MFE01Δ*fliA+*EV (EV: empty vector) mutant strain, with relative transcription higher than that of the wild-type strain MFE01. This significant increase in expression of the *flgM*, *flaA*, *fliS*, and *motA* genes in MFE01Δ*fliA*+*fliA* confirms that transcription of these genes was controlled by the FliA factor. We conclude that FliA activates transcription of these flagellar class IV genes in the MFE01 strain.

### 3.5. fliA Overexpression Restores Motility and Mucoidy in MFE01Δhcp1 and MFE01ΔtssC Mutants

**Figure 5:**
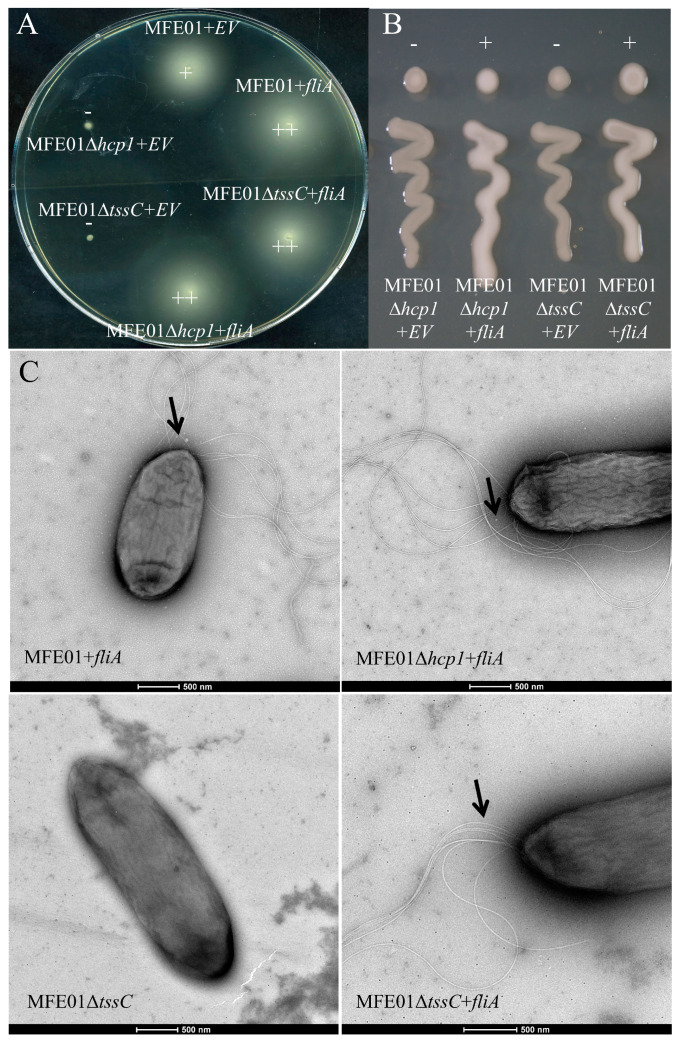
Effect of introducing *fliA* on phenotypes in MFE01 and derivatives. (**A**) Impact of introducing *fliA* on the swimming motility of MFE01 and derivatives. Swimming assays were performed on 0.3% LB agar for 24 h at 28 °C. +: motile, −: non-motile, ++: hyper-motile, EV: empty pPSV35 vector. The images shown are representative of three assays (*n* = 3). (**B**) Mucoid phenotypes after introducing *fliA* into MFE01 and derivatives. Mucoidy was assessed on 1.5% LB agar, supplemented with 50 µg/mL gentamycin, after incubation for 24 h at 28 °C. EV: empty pPSV35 vector, +: mucoid, −: non-mucoid. The images shown are representative of three assays (*n* = 3). (**C**) Flagella of *P. fluorescens* MFE01 and derivatives after introducing *fliA*. Transmission electron microscopy images of bacteria grown at 28 °C (negative stain). MFE01Δ*tssC* contained the empty pPSV35 vector. Arrows indicate multiple flagella. These images are representative of at least 35 bacteria for each strain.

We thus introduced the *fliA* gene in *trans* in various mutants. Overexpression of *fliA* restored both the motile and mucoid phenotypes in the MFE01Δ*tssC* and MFE01Δ*hcp1* mutants (Figure 5A,B) and FliA overproduction conferred a hyper-motile phenotype in both mutants as well as the wild-type strain (Figure 5A). Transmission electron microscopy confirmed an increase in the number of flagella upon *fliA* overexpression in MFE01, MFE01Δ*hcp1+fliA,* or MFE01Δ*tssC+fliA* (Figure 5C). Overexpression of *fliA* in *trans* promoted strong expression of class IV genes, possibly explaining the hyper-motile phenotype observed for MFE01Δ*fliA*+*fliA*. The restoration of the motile phenotype seems to indicate that the flagellar defect observed in T6SS mutants is due to perturbations independent of class I, II, or III flagellar gene expression and occurs after switching of the export substrate from rod-and-hook components to filament proteins [56]. This suggests specific perturbation of class IV gene expression during an Hcp1-T6SS imbalance. 

### 3.6. Class IV Genes Expression is Affected in MFE01Δhcp1 but not in fliA Transcription

**Figure 6:**
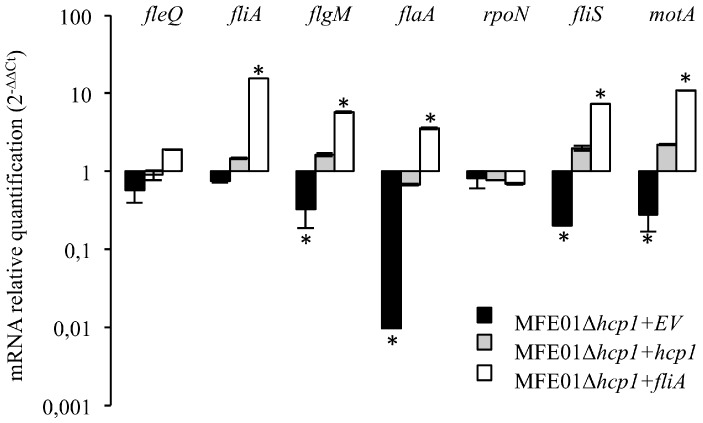
Expression of flagellar genes in various MFE01 mutants. The mRNA levels shown are relative to those obtained in MFE01 carrying the empty pPSV35 vector. Relative levels of gene expression are based on the comparative CT (2^−ΔΔCT^) method, using MFE01 *recA* mRNA as endogenous control. Statistical analyses were performed using Non-parametric Mann–Whitney Tests (two tailed). A *p*-value < 0.05 was considered to be statistically significant. * *p* < 0.05, *n* = 6. Data shown represent the mean ± SEM.

We performed qRT-PCR in MFE01Δ*hcp1*, MFE01Δ*hcp1+hcp1*, and MFE01Δ*hcp1+fliA* to examine the flagellar gene expression during an Hcp1-T6SS imbalance (Figure 6). The expression of the *fleQ* and *rpoN* genes was not significantly affected in MFE01Δ*hcp1*, suggesting that disruption of Hcp1-T6SS has no impact on the transcription of factors controlling class I, II, and III flagellar gene expression. We assessed whether flagellar class IV gene expression was affected in MFE01Δ*hcp1.* Disruption of *hcp1* significantly reduced the transcription of the *flaA, flgM, fliS*, and *motA* genes, relative to that in MFE01. Introduction of the *hcp1* gene in *trans* restored their transcription, consistent with the recovery of motility of MFE01Δ*hcp1*+*hcp1*. Moreover, the profile of flagellar class IV gene expression in MFE01Δ*hcp1* was comparable to that of MFE01Δ*fliA* (Figure 4C). Surprisingly, we observed no decrease of *fliA* transcription in MFE01Δ*hcp1* to explain the decrease of flagellar class IV gene expression. 

This result is not concordant with the decreased *fliA* transcription observed in T6SS mutants in *E. coli* and *Ralstonia solanacearum* [45,46]. No data are available for the study concerning *Citrobacter freundii* on the effect of the *tssM* mutation on *fliA* transcription [44]. We then reintroduced *fliA* in *trans* into MFE01Δ*hcp1*, resulting in higher class IV gene expression than in the wild-type strain. These results, coupled with the restoration of motility in MFE01Δ*hcp1* by the overexpression of *fliA,* suggest specific perturbation of class IV gene transcription upon the disruption of *hcp1*, without a decrease in *fliA* transcription. This implies that the FliA protein would be not available for class IV gene transcription in MFE01Δ*hcp1,* despite an equivalent level of transcription of *fliA*. It is possible that a protein that specifically interacts with FliA may accumulate in the cytoplasm of MFE01Δ*hcp1*, thus preventing the RNA polymerase from transcribing class IV flagellar genes.

### 3.7. FlgM is not Secreted in MFE01Δhcp1

In *P. aeruginosa*, the anti-sigma factor FlgM negatively regulates class IV gene expression, interacting directly with FliA [40]. Such sequestration of FliA by FlgM leaves no free FliA available for class IV gene expression. To obtain a functional flagella, the switch between the expression of class III to class IV genes occurs when the hook achieves the correct length (for details see Figure S1). Upon proper assembly of the flagellar hook basal body, FlgM is secreted through the incomplete flagellar apparatus, releasing the sigma factor FliA. Free FliA interacts with RNA polymerase and promotes class IV gene expression. As *flgM* gene expression depends on FliA and FlgM protein interacts directly with this sigma factor, FlgM exerts negative feedback regulation on *flgM* transcription.

**Figure 7:**
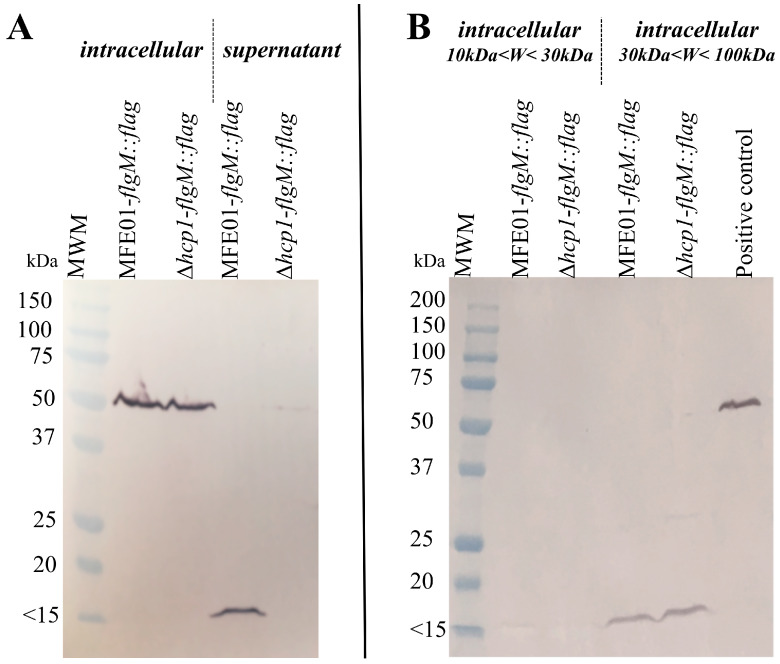
Localisation of FlgM::Flag in MFE01 or MFE01Δ*hcp1*. (**A**) Western-blot analysis of protein fractions of MFE01-*flgM::flag* and MFE01Δ*hcp1*-*flgM::flag*. An anti-Flag antibody coupled to alkaline phosphatase was used to visualise FlgM-Flag proteins. The images shown are representative of three assays (*n* = 3). The band at 50 kDa corresponds to MFE01 intracellular alkaline phosphatase. The band with a molecular weight < 15 kDa (dye front) corresponds to FlgM-Flag (11 kDa). (**B**) Western-blot analysis of concentrated intracellular protein fractions of MFE01- *flgM::flag* and MFE01Δ*hcp1*-*flgM::flag.* An anti-Flag antibody coupled to alkaline phosphatase was used to visualise FlgM-Flag proteins. The images shown are representative of three assays (*n* = 3). The positive control is a non-concentrated intracellular fraction in which the band at 50 kDa corresponds to MFE01 periplasmic alkaline phosphatase. The band with a molecular weight < 15 kDa (dye front) corresponds to FlgM-Flag (11 kDa).

According to the decrease of *flgM* transcription in MFE01Δ*hcp1* (Figure 6), reduction of FlgM was expected in this mutant. To obtain information on the quantity of FlgM, we introduced a chromosomal translational fusion of the *flag* sequence (encoding the DYKDDDDK peptide) with the *flgM* gene into MFE01 and MFE01Δ*hcp1*. We analysed supernatant and intracellular proteins of these strains by western blotting using an anti-Flag antibody coupled to alkaline phosphatase to visualise the FlgM-Flag proteins. MFE01-*flgM::flag* was able to secrete the FlgM anti-sigma factor, in contrast to MFE01Δ*hcp1*-*flgM::flag* (Figure 7A). This result was confirmed by proteomics analysis of MFE01 and MFE01Δ*hcp1* culture supernatants. FlgM was only identified in the wild-type supernatant, using our identify filter. We conclude that the anti-sigma factor FlgM is not secreted by MFE01Δ*hcp1* and may bind to free FliA in the cytoplasm. In the intracellular fraction, the band of 50 kDa corresponding to MFE01 periplasmic alkaline phosphatase was used as positive control and FlgM-Flag was not detected. It is possible that the FlgM-Flag proteins concentration under this condition was below the detection threshold.

We thus concentrated the intracellular proteins of the MFE01-*flgM::flag* and MFE01Δ*hcp1*-*flgM::flag* strains under native conditions using two membrane cut-off values, resulting in a 10 to 30 kDa fraction and a 30 to 100 kDa fraction. We chose these native conditions to explore the formation of FlgM complexes with other proteins. The fractions were then analysed by western blotting after SDS-PAGE (Figure 7B). In these fractions the MFE01 periplasmic alkaline phosphatase, which is associated with cell debris, was not detected. FlgM was recovered in both strains in the fraction of intracellular protein complexes of 30–100 kDa, whereas the molecular weight of FlgM is 11 kDa. FlgM appears to be associated with one or more other proteins in the intracellular fraction. In MFE01Δ*hcp1*, *flgM* transcription (which is FliA dependent) was lower than that in MFE01. However, by western-blot analysis, the quantity of intracellular FlgM protein appeared to be equivalent in MFE01Δ*hcp1* than in MFE01. FlgM, which is not secreted by MFE01Δ*hcp1*, may accumulate in this mutant and thus inhibit FliA-mediated class IV flagellar gene transcription.

These results are in line with the reduced transcription of the *flgM* gene in MFE01Δ*hcp1* (Figure 6), which decrease the quantity of FlgM protein in this mutant. Indeed, if we add the amount of FlgM secreted and the amount of intracellular FlgM, there is a higher production of FlgM in the wild-type strain.

### 3.8. FlgM Overexpression Perturbs MFE01 and MFN1032 Motility

**Figure 8:**
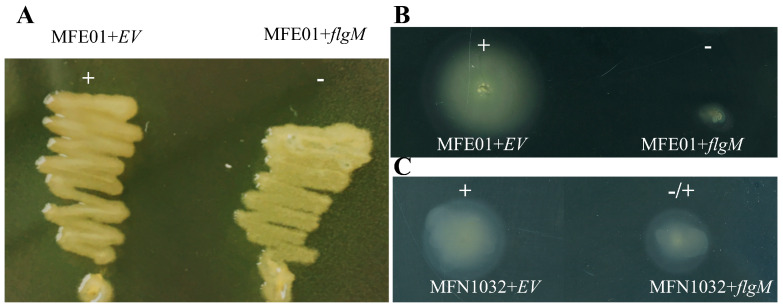
Phenotypic effects of *flgM* overexpression in MFE01 and MFN1032. (**A**) Mucoid phenotype of MFE01+*flgM*. Mucoidy was assessed on 1.5% LB-agar, supplemented with 50 µg/mL gentamycin, after incubation for 24 h at 28 °C. EV: empty pJN105 vector, +: mucoid, −: non-mucoid. The images shown are representative of three assays (*n* = 3). (**B**) Swimming motility of MFE01+*flgM*. Swimming assays were performed on 0.3% LB-agar, supplemented with 1% arabinose and 50 mg/mL gentamycin at 28 °C. (*n* = 3). −: non-motile, +: motile, EV: empty pJN105 vector. The images shown are representative of three assays. C. Swimming motility of MFN1032+*flgM*. Swimming assays were performed on 0.3% LB-agar, supplemented with 1% arabinose and 50 mg/mL gentamycin at 28 °C. −: non-motile, +: motile. EV: empty pJN105 vector. The images shown are representative of three assays.

We tested our hypothesis of FlgM accumulation in MFE01Δ*hcp1* by introducing *flgM* in *trans* under an inducible arabinose promoter into MFE01 (Figure 8A,B). Overproduction of FlgM in MFE01+*flgM* resulted in attenuated mucoid (Figure 8A) and non-motile (Figure 8B) phenotypes. These results are consistent with the accumulation of FlgM in MFE01Δ*hcp1* leading to FliA sequestration and inhibition of its transcriptional activity.

Previously, we demonstrated that MFE01 is able to immobilise the prey strain MFN1032, a clinical isolate of *P. fluorescens* [9], via Hcp1-T6SS. We explored the role of FlgM in this case of inhibiting the motility of prey cells by introducing the plasmid carrying the MFE01 *flgM* gene into the MFN1032 strain. Expression of *flgM* in MFN1032 resulted in a decrease in the swimming motility of this strain (Figure 8C).

## 4. Conclusions

Here, we investigated the correlation between T6SS, flagella and mucoidy in MFE01. Inactivation of Hcp1 production or T6SS activity renders this strain non-motile and non-mucoid. We demonstrate that these two phenotypes are sigma factor FliA dependent and that mutation of *hcp1* resulted in lower FliA transcription dependent activity without a decrease in *fliA* gene transcription. This may be explained by the sequestration of FliA by its anti-sigma factor FlgM. Indeed, FlgM was not secreted by the MFE01Δ*hcp1* mutant and appeared to accumulate in the bacteria. Finally, FlgM was able to decrease flagellar motility in the prey strain of the same species. These results led us to formulate two hypotheses: (i) the anti-sigma factor FlgM may be secreted through Hcp1-T6SS in MFE01 and accumulate in the T6SS mutant or (ii) a toxin secreted through Hcp1-T6SS and involved in decreasing the motility of prey cells may accumulate in the MFE01 T6SS mutants and stabilise the FliA/FlgM complex. Interactome studies with the FlgM and Hcp1 proteins are in progress to test these hypotheses.

Elucidation of the mechanism of crosstalk between Hcp1-T6SS and motility would be a strategic weapon in the protection of plants. Indeed, several *Pseudomonas* strains have been shown to be potential biocontrol agents. For example, *P. fluorescens* MFE01 and *P. putida* KT2440 can protect plants in a T6SS-dependant manner against several phytopathogens, including *Pectobacterium atrosepticum* and *Xanthomonas campestris* under *in planta* conditions [33,57]. In these studies, the identified effectors were antibacterial toxins, but other toxins able to limit phytopathogen motility may be involved in plant protection. Such toxins could be more environmentally friendly than antibacterial toxins by preventing phytopathogen plant colonisation without destabilising the natural balance of the plant microbiota.

## Figures and Tables

**Table 1:** Strains and plasmids.

Strain or Plasmid	Relevant Characteristics	Reference/Source
*Pseudomonas fluorescens*		
MFE01	Air isolate, Rif^R^	[33]
MFE01+pPSV35	MFE01 with pPSV35 empty vector, Gm^R^	[33]
MFE01+*fliA*	MFE01 with pPSV35 carrying wild-type *fliA* gene, Gm^R^	This study
MFE01Δ*tssC*	MFE01 with a in *frame* central deletion in *tssC* gene	[9]
MFE01Δ*tssC*+pPSV35	MFE01Δ*tssC* with pPSV35 empty vector, Gm^R^	[34]
MFE01Δ*tssC*+*tssC*	MFE01 with pPSV35 carrying wild-type *tssC* gene, Gm^R^	[34]
MFE01Δ*tssC*-R	MFE01Δ*tssC* with chromosomal introduction of wild-type *tssC* gene	This study
MFE01Δ*tssC*+*fliA*	MFE01Δ*tssC* with pPSV35 carrying wild-type *fliA* gene, Gm^R^	This study
MFE01Δ*hcp1*	MFE01 with *hcp1* gene disruption, Tc^R^	[9]
MFE01Δ*hcp1*+pPSV35	MFE01Δ*hcp1* with pPSV35 empty vector, Tc^R^, Gm^R^	[9]
MFE01Δ*hcp1*+*hcp1*	MFE01Δ*hcp1* with pPSV35 carrying wild-type *hcp1* gene, Tc^R^, Gm^R^	[9]
MFE01Δ*hcp1*+*fliA*	MFE01Δ*hcp1* with pPSV35 carrying wild-type *fliA* gene, Tc^R^, Gm^R^	This study
MFE01Δ*hcp2*	MFE01 with early stop codon in *hcp2* gene	[33]
MFE01Δ*hcp2*+pPSV35	MFE01Δ*hcp2* with pPSV35 empty vector, Gm^R^	This study
MFE01Δ*hcp3*	MFE01 with *in frame* deletion in *hcp3* gene	[34]
MFE01Δ*hcp3*+pPSV35	MFE01Δ*hcp3* with pPSV35 empty vector, Gm^R^	[34]
MFE01Δ*fliA*	MFE01 with in *frame* central deletion in *fliA* gene	This study
MFE01Δ*fliA*+pPSV35	MFE01Δ*fliA* with pPSV35 empty vector, Gm^R^	This study
MFE01Δ*fliA*+*fliA*	MFE01Δ*fliA* with pPSV35 carrying wild-type *fliA* gene, Gm^R^	This study
MFE01+pJN105	MFE01 with pJN105 empty vector, Gm^R^	This study
MFE01+*flgM*	MFE01 with pJN105 carrying wild-type *flgM* gene, Gm^R^	This study
MFE01-*flgM::flag*	MFE01 with 3’ *flgM*::*flag* transcriptional *fusion*	This study
MFE01Δ*hcp1*-*flgM::flag*	MFE01Δ*hcp1* with 3’ *flgM::flag* transcriptional fusion	This study
MFN1032	Clinical isolate	[35]
MFN1032+pJN105	MFN1032 with pJN105 empty vector, Gm^R^	This study
MFN1032+*flgM*	MFN1032 with pJN105 carrying wild-type *flgM* gene, Gm^R^	This study
***Escherichia coli***		
S17.1	RP4-2-Tc::Mu, *aph*::Tn7, *recA*, Sm^R^, donor strain for conjugation	[36]
Top10®	F- *mcrA* Δ(*mrr-hsd*RMS-*mcr*BC) Φ80*lacZ*ΔM15 Δ*lac*X74 *recA*1 *araD*139 Δ(*araleu*)7697 *galU galK rpsL* (StrR) *endA1 nupG*	ThermoFischer Scientific
Plasmids		
pPSV35	*Pseudomonas aeruginosa oriV*, *lacI^q^ mob*+, P*lac*UV5, pUC18MCS, expression vector, Gm^R^	[37]
pAKE604	Conjugative suicide vector, *oriT*, *lacZ*, *sacB*, Ap^R^, Km^R^	[38]
pJN105	Arabinose-inducible expression plasmid, Gm^R^	[39]

**Table 2:** Oligonucleotides used in this study. The underlined sequences correspond to the *flag* sequence.

**Mutagenesis Primers**	**Primer Sequence** (**5’--> 3’**)
Muta1-fliA-F	ACACTGGCCGACGTTATC
Muta2-fliA-EcoRI-R	TAATAAGAATTCGTAAAGATTCATGCCACTGG
Muta3-fliA-EcoRI-F	TAATAAGAATTCCTGTTCAGTTTCGACGAC
Muta4-fliA-R	CTTCAGCAGTCACCATCAA
Muta1-3’flag-flgM	TGATCAGGTCATCACACTG
Muta2-3’flag-flgM	CTTGTCATCGTCATCTTTATAATCGCGCTGGGCTTCGAAGTTG
Muta3-3’flag-flgM	GATTATAAAGATGACGATGACAAGTAGGCTTTTGCCGGCGCCAG
Muta4-3’flag-flgM	TTCATGGAAGGTGATGATCA
Muta1-tssC-F	CTGAGACTCCAGTAGCCAAG
Muta4-tssC-R	ATGTCATTGAGATCGGGCAA
**Surexpression primers**	**Primer sequence** (**5’--> 3’**)
fliA-EcoRI-F	TAATAAGAATTCGGCATCTGGAATTTTTCGT
fliA-XbaI-R	TAATAATCTAGATCCCCACACTGCCTTCA
flgM-EcoRI-F	TAATAAGAATTCTCCAAATTCCCAGAGGTTTT
flgM-XbaI-R	TAATAATCTAGAGTCGTTGATCAGTTGCAATA
**qPCR primers**	**Primer sequence** (**5’--> 3’**)
qRT-PCR-RecA-F	AAGGGTGCCGTAATGCGTAT
qRT-PCR-RecA-R	ATATCCAGACCCAGAGAGCCAGTA
qRT-PCR-FliA-F	CTGGTGTTGGCGCTGTACTAC
qRT-PCR-FliA-R	GCCAAGGACTTCACCGATTT
qRT-PCR-FlgM-F	GTACCAGCAACGCCAAGGAA
qRT-PCR-FlgM-R	TGTACCGACTCCCCGCTTT
qRT-PCR-FleQ-F	CATCGCGAACCCAATCTGT
qRT-PCR-FleQ-R	GGCCACTTGCTGCATCATCT
qRT-PCR-RpoN-F	ACTGGTCGCAGCGGAAAAT
qRT-PCR-RpoN-R	ATGCCTTGTGCCTCCAGTAAA
qRT-PCR-FliS-F	GATGTTAGCCCTTCGGCAGTAC
qRT-PCR-FliS-R	CACCTTCCATCAACATTTGCA
qRT-PCR-FlaA-F	ACACCCAGGCCATCCAGAA
qRT-PCR-FlaA-R	TGCAGGATGTCGGTCGAA
qRT-PCR-MotA-F	GCGTTCGTCTGCGATTACCT
qRT-PCR-MotA-R	CGTGCGGAGCCATGTTG

## Supplementary Materials

The following are available online at https://www.mdpi.com/2076-2607/8/5/622/s1. Figure S1. Flagellar assembly in *Pseudomonas*. This scheme is based on the literature [29,31,47,51,52]. Unfolded proteins are represented as curved lines, of which the colour is specific for each protein. Indeed, the proteins are unfolded before export. (1) Assembly of the flagellar hook (requiring cap protein FlgD): Anti-sigma factor FlgM sequesters FliA, inhibiting class IV flagellar gene transcription. (2) The hook is formed and its length is controlled by the protein FliK (the hook reaches its mature length of ∼55 nm). The FlgD protein is released and FlgM is secreted into the extracellular medium, thus releasing the sigma factor FliA. This enables class IV regulon transcription. (3) The FlgN chaperone allows secretion of the junction-proteins (hook-filament), FlgK and FlgL. (4) The cap protein FliD, secreted with the help of its chaperone FliT, forms a hexamer (in *Pseudomonas aeruginosa*). (5) The flagellar filament, consisting of flagellin FlaA (or FliC), during the assembly phase. The chaperone FliS allows flagellin and FlaG (control of filament length) secretion. (6) Completion of filament assembly. (7) The MotA and MotB proteins, which allow proton influx (proton motive-force), form a heterodimer. FliA also controls the transcription of several other genes, such as *flgZ* and chemotaxis genes (not represented in this figure). Table S1. The functions of genes mentioned in this work.
