# Supplementary material for: Crosstalk between the Type VI Secretion System and the Expression of Class IV Flagellar Genes in the Pseudomonas fluorescens MFE01 Strain
Source: Microorganisms. 2020 Apr 25;8(5):622. doi: 10.3390/microorganisms8050622 (PMC7286023; doi:10.3390/microorganisms8050622)
Supplement: Supplementary file 1 [file microorganisms-08-00622-s001.zip › Figure S1 et table S1.docx]

**Figure S1. Flagellar assembly in *Pseudomonas.***

This scheme is based on the literature [29,31,47,51,52]. Unfolded proteins are represented as curved lines, of which the colour is specific for each protein. Indeed, the proteins are unfolded before export. 1) Assembly of the flagellar hook (requiring cap protein FlgD): Anti-sigma factor FlgM sequesters FliA, inhibiting class IV flagellar gene transcription. 2) The hook is formed and its length is controlled by the protein FliK (the hook reaches its mature length of ∼55 nm). The FlgD protein is released and FlgM is secreted into the extracellular medium, thus releasing the sigma factor FliA. This enables class IV regulon transcription. 3) The FlgN chaperone allows secretion of the junction-proteins (hook-filament), FlgK and FlgL. 4) The cap protein FliD, secreted with the help of its chaperone FliT, forms a hexamer (in *Pseudomonas aeruginosa*). 5) The flagellar filament, consisting of flagellin FlaA (or FliC), during the assembly phase. The chaperone FliS allows flagellin and FlaG (control of filament length) secretion. 6) Completion of filament assembly. 7) The MotA and MotB proteins, which allow proton influx (proton motive-force), form a heterodimer. FliA also controls the transcription of several other genes, such as *flgZ* and chemotaxis genes (not represented in this figure)

**Table S1. The functions of genes mentioned in this work**

| **Gene** | **Protein function or localization** | **MFE01 GenbanK accession number** |
| --- | --- | --- |
| *fleQ* | RpoN dependent transcriptional activator, Master regulator, required for classes I and II flagellar genes expression | MT180957 |
| *rpoN* | σ^54^ sigma factor, required for classes II and III flagellar genes expression | MT180956 |
| *fliA* | σ^28^ sigma factor, required for class IV flagellar genes expression | MT180958 |
| *flgM* | σ^28^-specific anti-sigma factor, interacts directly with FliA | MT180962 |
| *flaA* | Flagellin, major filament component | MT180960 |
| *flaG* | Filament length control | MT180963 |
| *flgN* | Chaperone; export of junction proteins FlgK-FlgL, initiation of filament assembly | MT180968 |
| *fliD* | Filament cap protein, controls flagellin assembly | MT180964 |
| *fliS* | Chaperone, export of flagellin | MT180961 |
| *fliT* | Chaperone, export of filament cap protein FliD | MT180965 |
| *flgZ* | PilZ domain-containing protein, interact with stator protein, implicated in motility regulation | MT180969 |
| *motA* | Proton-dependent-stator complex, motor rotation | MT180966 |
| *motB* | Proton-dependent-stator complex, motor rotation | MT180967 |
